# Diagnostic Accuracy of Monofilament Tests for Detecting Diabetic Peripheral Neuropathy: A Systematic Review and Meta-Analysis

**DOI:** 10.1155/2017/8787261

**Published:** 2017-10-08

**Authors:** Fengyi Wang, Jiaqi Zhang, Jiadan Yu, Shaxin Liu, Rengang Zhang, Xichao Ma, Yonghong Yang, Pu Wang

**Affiliations:** ^1^Rehabilitation Medicine Center, West China Hospital of Sichuan University, Chengdu, China; ^2^West China School of Medicine, Sichuan University, Chengdu, China; ^3^Department of Rehabilitation Sciences, The Hong Kong Polytechnic University, Hung Hom, Hong Kong; ^4^Rui Jin Hospital, Shanghai Jiao Tong University School of Medicine, Shanghai, China

## Abstract

**Objective:**

To systematically evaluate the diagnostic accuracy of monofilament tests for detecting diabetic peripheral neuropathy.

**Methods:**

We searched EMBASE (OvidSP), MEDLINE (OvidSP), the Cochrane Library, and Web of Science to identify diagnostic accuracy trials of monofilament tests for detecting diabetic peripheral neuropathy. We used a hierarchical summary receiver operating characteristics (HSROC) model to conduct the meta-analysis of diagnostic accuracy of monofilament tests for detecting diabetic peripheral neuropathy.

**Results:**

A total of 19 comparative trials met the inclusion criteria and were part of the qualitative synthesis. Eight trials using nerve conduction studies as the reference standard were selected for the meta-analysis. The pooled sensitivity and specificity of monofilament tests for detecting diabetic peripheral neuropathy were 0.53 (95% confidence interval (CI) 0.32 to 0.74) and 0.88 (95% CI 0.78 to 0.94), respectively. The pooled positive likelihood ratio and negative likelihood ratio were 4.56 (95% CI 2.93 to 7.10) and 0.53 (95% CI 0.35 to 0.81), respectively.

**Conclusions:**

Our review indicated that monofilament tests had limited sensitivity for screening diabetic peripheral neuropathy. The clinical use of the monofilament test in the evaluation of diabetic peripheral neuropathy cannot be encouraged based on currently available evidence.

## 1. Introduction

Diabetes mellitus (DM) is one of the most common metabolic diseases worldwide. The incidence, prevalence, and importance of DM as a chronic disease are increasing [1]. In 2010, approximately 21 million US adults aged 20 years or older had total confirmed diabetes (i.e., self-reported diabetes or diagnostic levels for both fasting glucose and calibrated HbA1c) [2]. Diabetic peripheral neuropathy (DPN) is one of the most common complications of DM. This problem is frequently associated with a loss of sensation in the foot and an increased incidence of foot ulcers [3, 4], resulting in foot infection and even amputation in individuals with DM in the late stage [5].

Early detection of DPN contributes to preventing of foot ulcers and amputations. Several methods are used to detect DPN, including quantitative sensory testing, physical examination scoring systems (e.g., the neuropathy disability score), nerve conduction studies (NCS), and electrodiagnostic tests [6–8]. NCS is regarded as the standard for DPN diagnosis [9, 10]; however, this examination is too time-consuming, highly demanding, and expensive to be implemented in many primary care settings. Thus, a portable, reliable, and valid tool for detecting DPN is urgently needed.

Monofilament tests have been widely used in clinical practice for DPN screening owing to their availability and convenience [11]. As a quantitative sensory test, a monofilament is used to test a single point of touch pressure. A 5.07/10 g monofilament is used to screen for the presence or absence of protective sensation [12, 13]. Several studies have explored whether a monofilament test is a useful screening tool for the early detection of DPN. Therefore, we conducted this systematic review with meta-analysis to quantitatively evaluate the currently available evidence regarding the diagnostic accuracy of monofilament tests for DPN detection.

## 2. Methods

### 2.1. Data Sources and Searches

We searched EMBASE (OvidSP, 1976 to April 2016), MEDLINE (OvidSP, 1946 to April 2016), the Cochrane Library (issue 4, 2016), and Web of Science (1995 to April 2016) to identify diagnostic accuracy studies of monofilament tests for detecting DPN. Our search strategy was focused on monofilament tests, diabetic peripheral neuropathy, and diagnostic accuracy. See Supplementary Material Appendices 1–4 available online at https://doi.org/10.1155/2017/8787261 for a complete list of search strategies.

### 2.2. Selection of Studies

One author screened all titles and abstracts generated by the electronic database searches for relevance and selected all potentially eligible studies for review of the full-text articles. Two reviewers independently assessed the full-text articles according to the inclusion criteria and exclusion criteria. When it was necessary, a third arbitrator resolved any disagreements that remained after discussion between the two reviewers. The inclusion criteria were as follows: (1) the study examined the diagnostic accuracy of a monofilament test for detecting DPN, (2) the article was published in English, and (3) the study provided sufficient data. The exclusion criteria were as follows: (1) the study was performed on patients without DM or (2) the study was performed on patients who had visible ulcers.

### 2.3. Data Extraction

Two authors independently extracted the following data from each included study: first author, year of publication, sample size, mean age of the participants, description of the monofilament, sites and number, threshold of the monofilament test, reference standard, sensitivity, and specificity. A third arbitrator resolved any remaining disagreements that the two review authors could not resolve through discussion. If more than one threshold was published in primary studies, we reported the diagnostic accuracy under all thresholds. The present systematic review of all diagnostic accuracy studies for the monofilament was conducted irrespective of reference standard utilized, whereas the quantitative synthesis was confined to trials using NCS as the reference standard. Additional information was extracted from studies that used NCS as the reference standard to demonstrate the variation across each study.

### 2.4. Assessment of Methodological Quality

We assessed the methodological quality of the studies using Quality Assessment of Diagnostic Accuracy Studies (QUADAS-2; http://www.bris.ac.uk/quadas), which has been recommended by Cochrane to assess the quality of primary diagnostic accuracy studies [14]. QUADAS-2 consists of four domains: patient selection, index test, reference standard, and patient flow/timing. Each domain is assessed for any risk of bias; the first three domains are also assessed for any concerns regarding applicability (see Supplementary Material Appendix 5). The risk of bias and applicability was analyzed using RevMan 5.3.

### 2.5. Statistical Analysis

Because of varying reference standards enrolled in studies, we selected studies that used NCS as the reference standard for meta-analyses of its validity in diagnosing DPN [9, 10]. The data on true positives, true negatives, false positives, and false negatives were calculated based on the data reported by each original study. And the sensitivity, specificity, diagnostic odds ratio, positive likelihood ratio (LR+), negative likelihood ratio (LR−), and their 95% confidence interval (CI) were presented in the forest plots performed by Meta-DiSc. The pooled sensitivity, specificity diagnostic odds ratio, positive likelihood ratio (LR+), and negative likelihood ratio (LR−) of included studies were calculated by using a hierarchical summary ROC model (HSROC) conducted by Stata 12.0.

## 3. Results

### 3.1. Results of the Search

**Figure 1:**
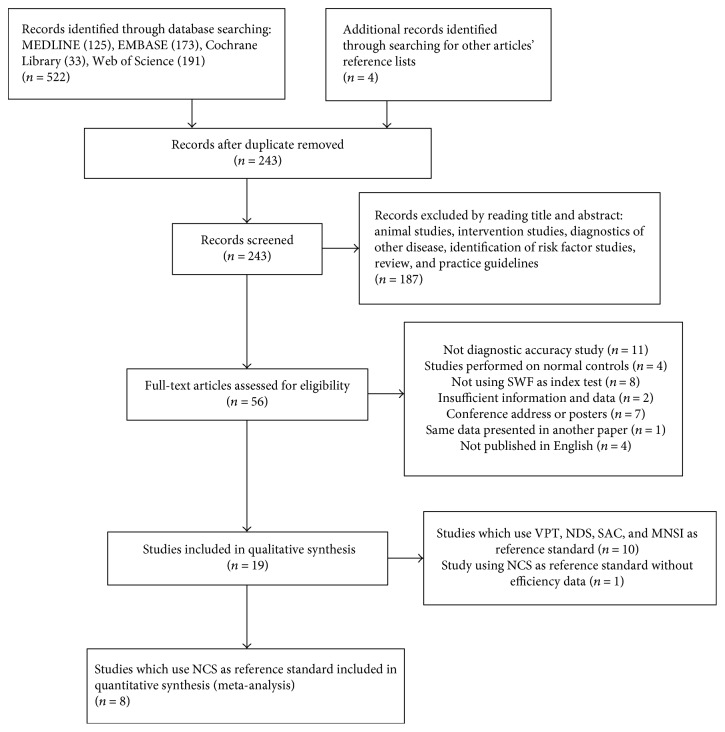
Flowchart of the study search and selection process.

A total of 522 records were identified through the electronic searches of MEDLINE (OvidSP) (*n* = 125), EMBASE (OvidSP) (*n* = 173), Cochrane (*n* = 33), and Web of Science (*n* = 191). We excluded 283 duplicate records. Four studies were identified by scanning the reference lists of the identified studies. A total of 187 irrelevant records were excluded by reading the titles and abstracts. In total, 56 potentially relevant studies were identified for full-text analysis. Of these, 11 studies were not diagnostic accuracy studies, 4 studies were performed on patients without DM, 8 studies did not use a monofilament test as the index test, 7 studies were conference address or posters, 2 studies were without sufficient information and data, another study used the same data set as another published paper, and 4 studies were not published in English. In total, 19 comparative studies [15–33] met all eligibility criteria and were selected for qualitative analysis. These studies were conducted from 1997 to 2015. See Figure 1 for the details of the study search and selection process.

### 3.2. Data Extraction and Management

Sensitivity and specificity were retrieved or calculated from data available in the primary studies. Tables 1 and 2 list the characteristics of the included studies. The total sample size of all 19 studies was 3566 subjects, and the mean age of the participants ranged from 42 to 65 years old. Of the 19 studies, 6 studies did not report the type of monofilament used and 13 clearly reported that Semmes-Weinstein monofilaments (SWF) were used as the index test tool. The most commonly used test site was the great toe (plantar surface or dorsal surface). Nine of the 19 studies used NCS as the reference standard; vibration perception threshold (VPT), neuropathy disability score, and the Michigan Neuropathy Screening Instrument (MNSI) were also used. In the 19 studies, the sensitivity of the monofilament test ranged from 0.06 to 0.99 and the specificity ranged from 0.455 to 1.00.  Table 3 shows the variations of the study set in the following: duration of DM, geographical distribution, and techniques of the monofilament test for studies using NCS as the reference standard.

### 3.3. Methodological Quality of the Included Studies

**Figure 2:**
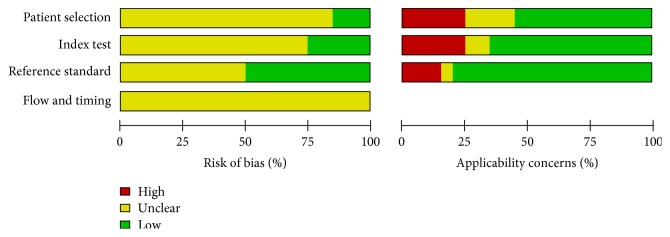
Risk of bias and applicability concerns: reviewers' judgments about each domain presented as percentages across included studies.

**Figure 3:**
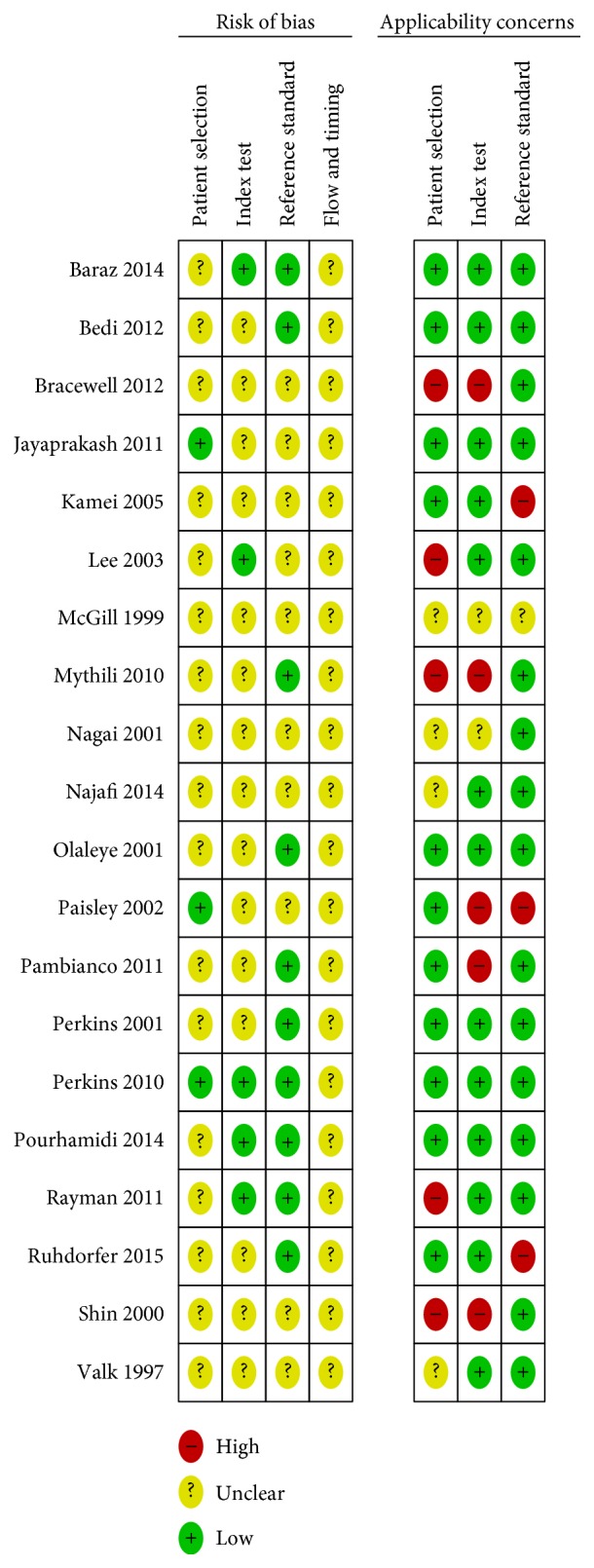
Risk of bias and applicability concerns: reviewers' judgments about each domain for each included study.

We assessed the methodological quality of the studies using QUADAS-2. The risk of bias and applicability concerns were analyzed using RevMan 5.3. Figures 2 and 3 show our assessment of each domain's risk of bias and applicability concerns for the included studies. In the selection of patients, three studies (Jayaprakash et al. [20], Paisley et al. [18], and Perkins et al. [29]) were labeled as low risk. The risk in the other 17 studies was unclear because they did not explicitly state whether a consecutive or random sample of patients was recruited or that the appropriate exclusions had been made.

Only five studies (Baraz et al. [32], Lee et al. [27], Perkins et al. [29], Pourhamidi et al. [31], and Rayman et al. [21]) had both a low risk of bias and low applicability concerns regarding the index test. It was unclear whether the index test results were interpreted without knowledge of the reference standard results in the other 15 studies. The reference standard domain in 10 studies (Bracewell et al. [23], Jayaprakash et al. [20], Kamei et al. [19], Lee et al. [27], McGill et al. [16], Nagai et al. [17], Najafi et al. [24], Paisley et al. [18], Shin et al. [34], and Valk et al. [15]) was unclear because the studies did not state whether the investigators performing the reference standard were blinded to the results of the index test (SWF). The risks for flow and timing domain in all studies were also unclear because either the interval between the index test and the reference standard was unknown or not all patients were included in the final analysis.

### 3.4. Meta-Analysis

Studies using NCS as the reference standard were included in the quantitative synthesis; however, one study (Ruhdorfer et al. [33]) was excluded because of insufficient data. The remaining eight studies included in the statistical analysis had a total of 1377 participants, with a sample size ranging from 37 to 478. Multiple thresholds were set in two studies (Olaleye et al. [25] and Baraz et al. [32]); we selected only the threshold that was closest to the threshold value (“half cutoff” threshold) used in other studies for the meta-analysis; therefore, a total of 12 groups of data were part of the meta-analysis.

**Figure 4:**
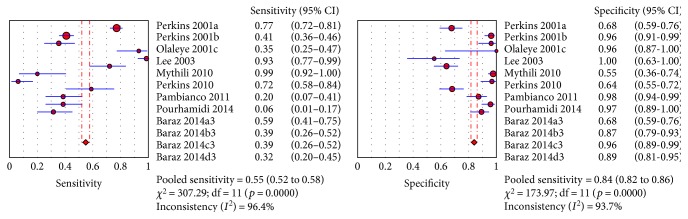
Forest plot of the sensitivity and specificity (red diamond) and its 95% CI (blue horizontal line).

**Figure 5:**
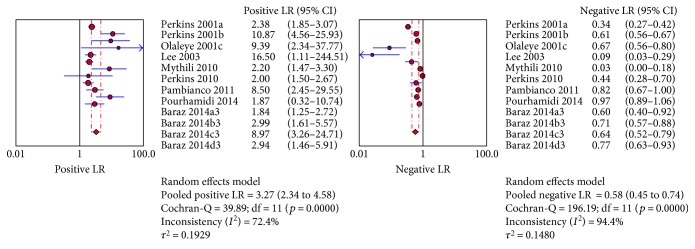
Forest plot of the summary LR+ and LR−.

**Figure 6:**
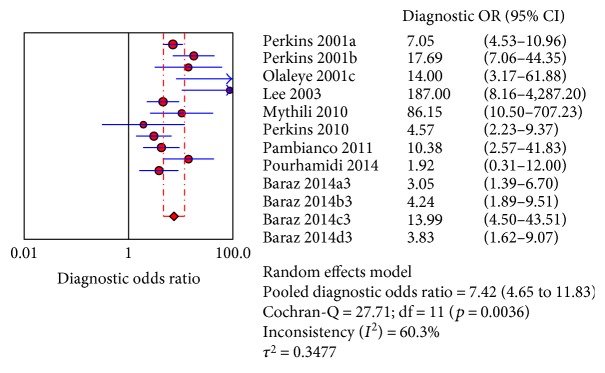
Forest plot of the diagnostic odds ratio.

**Figure 7:**
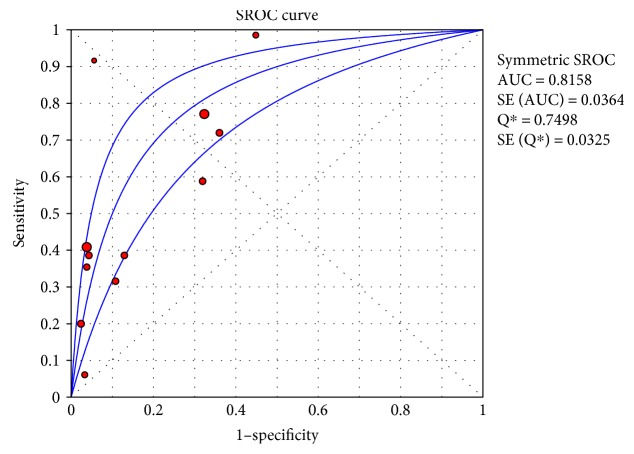
SROC with a 95% confidence interval for monofilament tests in the diagnosis of DPN.

**Figure 8:**
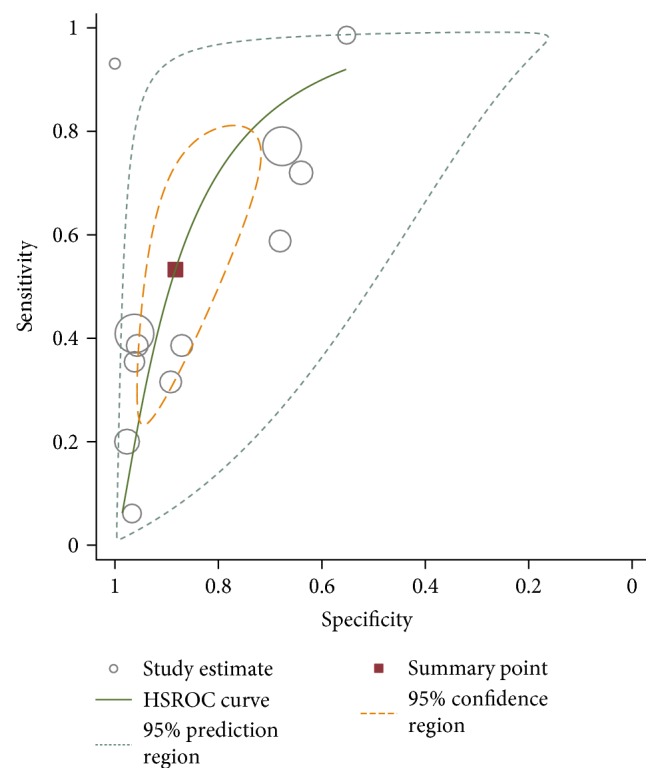
HSROC of the monofilament test for detecting DPN.

The sROC analysis for the studies yielded an overall weighted area under the curve of 0.8158 (0.0364); the index Q^∗^ value was 0.7498 (0.035), which is a strong indicator (0.7 < ROC = 0.8158< 0.9). A visual inspection of the forest plots shows large deviations, which indicate possible heterogeneity. Statistical tests such as chi-square, Cochran-Q, and the inconsistency index (*I*-squared) can be used to quantify the amount of heterogeneity (see Figures 4–6). The forest plots show increasing sensitivities with decreasing specificities. Examination of the sROC curves for the studies reveals a “shoulder arm” plot, which indicates a threshold effect (Figure 7). Hence, a HSROC model was performed to pool the diagnostic parameters while considering the threshold effect. Under the HSROC model, the pooled sensitivity of studies was 0.53 (95% CI 0.32 to 0.74) and the pooled specificity was 0.88 (95% CI 0.78 to 0.94). The pooled diagnostic odds ratio (DOR) of 8.62 (95% CI 4.69 to 15.84). The pooled positive likelihood ratio (LR+) and negative likelihood ratio (LR−) values were 4.56 (95% CI 2.93 to 7.10) and 0.53 (95% CI 0.35 to 0.81), respectively (Table 4, Figure 8).

Figures 4–6 showed the forest plot of the sensitivity, specificity, diagnostic odds ratio, LR+, and LR−, and the SROC and HSROC plot were presented in Figure 7 and Figure 8, respectively.

## 4. Discussion

Regular sensory examinations for people with DM are recommended by clinical guidelines [35]. Therefore, a portable and useful screen is necessary. Monofilament tests are one of the most common screening tools for DPN in primary care settings due to their availability. However, we found many factors for consideration in the application of monofilament tests, and the role of monofilament tests in DPN diagnosis needs to be clarified.

A previous review [36] reported that the use of monofilament testing for the diagnosis of peripheral neuropathy has low sensitivity, which is in accordance with the main results of our study. Because of the variability of testing procedures, reference standards, and the application of the monofilament (number, site, and definition of thresholds), quantitative analysis was seldom used in previous reviews. We found a strong threshold effect among studies; therefore, the HSROC model was enrolled in the present study for pooled analyses, which jointly summarize pooled sensitivity and specificity while taking into account the threshold effect. When compared with NCS, our meta-analysis demonstrates that monofilament tests are fairly accurate for diagnosing DPN in individuals with DM. The pooled sensitivity and specificity values for studies using NCS as a reference standard were 0.53 and 0.88, respectively; thus, the sole use of a monofilament test to screen for DPN cannot be recommended at this stage because of the test's low sensitivity. The pooled LR+ and LR− values were 4.56 and 0.53, which are informative but not strong indicators for DPN confirmation or exclusion. A previous review [36] differed from the present review, in terms of their inclusion criteria, which was not limited to individuals with DM. For example, Shin et al. [34] enrolled patients who were referred to a foot clinic; this study was included in the previous review [36] but excluded by the present study because data that was specific to the cohorts of DM could not be extracted, and some studies [37–39] with healthy controls were excluded in our review as well. We also tried to exclude the studies of patients with visible ulcers, which can directly affect the diagnosis by monofilament tests or NCS; however, many studies did not report their inclusion criteria in detail to describe whether the participants have suffered visible foot ulcers or not.

Our findings are based on studies with low methodological quality (as identified by QUADAS-2); hence, many factors were labeled as being unclear. In practice, the monofilament test should directly follow or precede the reference standard test, with examiners blinded to the results. However, most of included studies did not report this information. An appropriate interval between the monofilament test and the reference standard is necessary because misclassification may occur due to recovery or deterioration of the targeted condition.

As part of a reliable clinical tool to assess changes in the protective sensation of the feet, the 5.07/10 g monofilament is the most commonly employed filament nowadays [40, 41]. However, Kamei et al. [19] suggested that SWF 4.31/2 g was a better diagnostic test for detecting DPN than 5.07/10 g monofilament, with sensitivity and specificity of 0.60 and 0.738, respectively. The monofilament test sites also varied considerably across studies. An 18-site sensory examination [42] using 10 g monofilament that results in sensory change at three or more sites indicates actual change in the protective sensation of the feet. The most commonly used sites are on the great toe (plantar and dorsal), but there is no evidence to confirm that this is the most sensitive location for testing. Additionally, there is not a protocol for the threshold set of monofilaments to screen DPN, including sites and numbers. In some studies, a monofilament test was performed on different sites with different cutoff points for positive results, with an increase of cutoff points to total points (e.g., 2/8 to 5/8); the sensitivity decreased, while the specificity increased, so the effects of bias should be considered. In addition, no clear difference in sensitivity was demonstrated by increasing the number of test points for DPN screening. In Baraz et al.'s study [32], the sensitivity of the monofilament at three and four points is almost similar to its sensitivity at eight and ten points.

At present, monofilament tests have been already widely used and advocated for in many clinical guidelines. However, there is no consensus on the optimal location, number of sites, and threshold values for DPN diagnosis. Therefore, further research is needed to standardize the method for clinical practice. As indicated by our meta-analysis, heterogeneity exists among studies. Two main causes are indicated: (1) there were different clinical protocols for the application of monofilaments in DM and (2) the subjects may differ in age, severity of DM, or other confounding factors. Semmes-Weinstein 5.07/10 g monofilaments (manufactured by North Coast Medical) were used in 13 of the 19 studies included in our review. The commercial manufacturing source was frequently identified in studies; however, the durability of monofilaments should be considered. Longevity and recovery testing suggest that each monofilament can be used with approximately 10 patients, with a period of 24 hours required between uses [43]. Additionally, changes in relative humidity and temperature may affect the physical properties of monofilaments [44]. These details were not provided in the included studies. In order to demonstrate the monofilament test's ability to guide clinical decision making and to improve patient outcome, the prognostic and predictive value of monofilament tests can and should be evaluated by diagnostic randomized controlled trial. However, this evidence is lacking as our research result.

The present review has three main limitations. Firstly, our meta-analysis was performed based on a small number of studies with obvious heterogeneity. Although we used a HSROC model in our analysis, our conclusion should be interpreted with caution. Secondly, the protocol for using monofilament tests in DPN screening varied from study to study, which made it difficult to draw a firm conclusion at this stage. Thirdly, restricting the search to English-language publications may result in missing some relevant literature. Last but not least, we did not search grey literature sources and it can be acknowledged as a potential source of publication bias.

## 5. Conclusion

In summary, our study found that the 5.07/10 g Semmes-Weinstein monofilament seemed to be a screen with limited sensitivity for DPN in primary care settings based on currently available evidence. Available studies with regard to the application of monofilament testing for DPN diagnosis varied greatly, and an optimal protocol for conducting monofilament tests in patients with DM is under exploration. Higher-quality studies on Semmes-Weinstein monofilament examination detecting DPN are needed.

## Supplementary Material

Appendix 1 Search strategy for EMBASE (OvidSP). Appendix 2 Search strategy for MEDLINE (OvidSP). Appendix 3 Search strategy for Cochrane. Appendix 4 Search strategy for Web of Science. Appendix 5 Assessment of methodological quality table QUADAS-2 tool.

## Figures and Tables

**Table 1:** The characteristics of the included studies using VPT, NDS, SAC, and MNSI as the reference standard.

Study (author year)	Sample size (male)	Age (years) Mean ± SD	Monofilament	Sites and number	Threshold	Reference test	Sensitivity (%) (95% CI)	Specificity (%) (95% CI)
Valk et al., 1997 [15]	68 (36)	51.6 ± NR	(a) SWF 5.07/10 g	Three sites on both feet: first toe, medial surface, and base of the third metatarsal bone	1 of 18	VPT	95.8	45.5
			(b) SWF 4.17/1 g					
			(c) SWF 6.10/75 g					
McGill et al., 1999 [16]	132 (NR)	57 (47.5–65.8)	MF 5.07/10 g	Five sites on the right foot: great toe, dorsum between the first and second metatarsals, the plantar aspect of the first metatarsal, the plantar aspect of the fifth metatarsal, the plantar aspect of the arch	(a) 5 of 5	VPT	(a) 31	(a) 100
					(b) 3 of 5		(b) 37	(b) 98
					(c) 1 of 5		(c) 39	(c) 83
Nagai et al., 2001 [17]	65 (NR)	61.0 ± 1.3	(a) SWF 5.07/10 g	Three sites on the foot: great toe, the plantar aspect of the first metatarsal, the plantar aspect of the fifth metatarsal	NR	Numbness in the toes and loss of ankle jerk and VPT	(a) 88	(a) 68
							(b) 85	(b) 73
			(b) SWF 4.56/4 g				(c) 48	(c) 86
			(c) SWF 4.31/2 g					
Paisley et al., 2002 [18]	124 (84)	55.4 ± 13.7	MF 5.07/10 g	Twice on the plantar surface of each hallux and also the 1st, 2nd, 3rd, and 5th metatarsal heads	3 of 10	(a) NDS	(a) 87.8	(a) 57.3
						(b) VPT	(b) 70	(b) 63.8
Kamei et al., 2005 [19]	82 (44)	61.6 ± 11.0	(a) SWF 5.07/10 g	The great toe, the plantar aspect of the first metatarsal, and site 3, the plantar aspect of the fifth metatarsal for each foot	NR	VPT	(a) 15–30	(a) 92.9
			(b) SWF 4.31/2 g				(b) 47.5–60	(b) 71.4–76.2
Jayaprakash et al., 2011 [20]	1044 (532)	53.3 ± 11.8	SWF 5.07/10 g	The plantar surface of great toe and base of first, third, and fifth metatarsals of both feet	1 of 8	VPT	63	93
Rayman et al., 2011 [21]	265	65 ± NR	MF 5.07/10 g	(a) 3 points in each foot: tips of the first, third, and fifth toes and dorsum of hallux of both feet	(a) 2 of 8	VPT	(a) 85	(a) 88
				(b) 4 points in each foot: tips of the first, third, and fifth toes	(b) 5 of 8		(b) 81	(b) 91
Bedi and Mittal, 2012 [22]	106 (48)	54.99 ± 11.08	SWF 5.07/10 g	The plantar surface of great toe and base of the first, third, and fifth metatarsals of both feet	1 of 8	VPT	48.9	48
Bracewell et al., 2012 [23]	141 (76)	56.9 ± 14.7	MF 5.07/10 g	Five sites: the 1st, 3rd, and 5th metatarsal heads on the plantar surface, the hallux pulp, the dorsal surface of the hallux proximal to the nail fold	NR	VPT	84	83
Najafi et al., 2014 [24]	107 (35)	57.6 ± 10.2	MF 5.07/10 g	The dorsum of the great toe midway between the nail fold and the DIP joint	3 of 10	MNSI	16.7	87

NR: not reported in the paper; MF: monofilament; SWF: Semmes-Weinstein monofilaments; VPT: vibration perception threshold; VDT: vibration detection thresholds; NDS: neuropathy disability score; MNSI: Michigan neuropathy screening instrument; SAC: San Antonio Consensus for DPN diagnosis: “1 of 6”: minimum of 6 points, 1 point reported as a positive result; DIP: distal interphalangeal joint.

**Table 2:** The characteristics of the included studies using NCS as the reference standard.

Study (Author Year)	Sample size (male)	Age (years) Mean ± SD	Monofilament	Prevalence of DPN	Sites and number	Threshold	Reference test	Sensitivity % (95% CI)	Specificity % (95% CI)
Olaleye et al., 2001 [25]	132 (84)	52.92 ± 11.4	SWF 5.07/10 g	59.8%	Noncallused site on the dorsum of the first toe just proximal to the nail bed and repeated four times on both feet	(a) 2 of 8	NCS	(a) 62	(a) 84
						(b) 3 of 8		(b) 58	(b) 92
						(c) 4 of 8		(c) 35	(c) 97
						(d) 5 of 8		(d) 30	(d) 97
Perkins et al., 2001 [26]	478 (319)	54 (NR)	SWF 5.07/10 g	72.2%	Noncallused site on the dorsum of the first toe just proximal to the nail bed and repeated four times on both feet	(a) 1 of 8	NCS	(a) 77	(a) 67.6
								(b) 40.8	(b) 96
						(b) 5 of 8			
Lee et al., 2003 [27]	37 (20)	57.0 ± 9.3	SWF 5.07/10 g	78.4%	The dorsal surface of the foot between the base of the first and second toes, the first, third, and fifth toes, the first, third, and fifth metatarsal heads, the medial and lateral midfoot, and the heel in random order	5 of 10	NCS	93.1	100
Mythili et al., 2010 [28]	100 (48)	52.9 (30–80)	SWF 5.07/10 g	71%	Both feet on the plantar surface of the hallux and centrally at the heel six times at each point	1 of 6	NCS	98.5	55
Perkins et al., 2010 [29]	175 (118)	57 ± 8	SWF 5.07/10 g	28.6%	Noncallused site on the dorsum of the great toe just proximal to the nail bed four times at each foot	5 of 8	NCS	72	64
Pambianco et al., 2011 [30]	195 (NR)	46.2 ± 7.2 (170) 41.4 ± 6.5 (25)	MF 5.07/10 g	12.8%	The dorsum of the great toe ten times for each foot	3 of 10	NCS	20	98
Pourhamidi et al., 2014 [31]	110 (61)	60 ± 1	SWF 5.07/10 g	44.5%	Three standard points (plantar surface of distal hallux and 1st and 5th metatarsal heads) bilaterally on the sole of the foot	1 of 6	NDS & NCS	6	97
Baraz et al., 2014 [32]	150 (47)	55.71 ± 8.95	SWF 5.07/10 g	38%	(a) Three points in each foot: the great toe, the plantar aspect of the first, and the fifth metatarsal head	(a1) 1 of 6	NCS	(a1) 53.8	(a1) 73.9
						(a2) 2 of 6		(a2) 43.6	(a2) 79.3
						(a3) 3 of 6		(a3) 35.9	(a3) 84.7
					(b) Four points in each foot: the plantar surface of hallux, and the first, third, and fifth metatarsal heads	(b1) 1 of 8		(b1) 51.3	(b1) 73
						(b2) 2 of 8		(b2) 46.2	(b2) 74.8
						(b3) 4 of 8		(b3) 38.5	(b3) 87.4
					(c) Eight points in each foot: the dorsal aspect of the first, second, third, fourth, and fifth digits; the dorsal aspect of the medial, central, and lateral aspect of mid foot	(c1) 1 of 16		(c1) 61.5	(c1) 77.5
						(c2) 2 of 16		(c2) 59	(c2) 79.3
						(c3) 8 of 16		(c3) 38.5	(c3) 95.5
					(d) 10 points in each foot: nine plantar sites (distal great toe, third toe, and fifth toe; first, third, and fifth metatarsal heads; medial foot, lateral foot, and heal) and one dorsal site	(d1) 1 of 20		(d1) 64.1	(d1) 64
						(d2) 2 of 20		(d2) 61.5	(d2) 64
						(d3) 10 of 20		(d3) 30.8	(d3) 89.2
Ruhdorfer et al., 2015 [33]	55 (28)	64.3 ± 12.6	SWF 5.07/10 g	70%	The big toe, the fifth toe, the heel, the arch of the foot, and on the dorsum of the foot	1 of 1	(a) SRS	(a) 76	(a) 79
							(b) NCS	(b) 67	(b) 67

NR: not reported in the paper; MF: monofilament; SWF: Semmes-Weinstein monofilaments; NCS: nerve conduction study; SRS: self-reported symptoms; “1 of 6”: minimum of 6 points, 1 point reported as a positive result.

**Table 3:** Moderator variables.

Study (author year)	Diabetes duration (years) Mean ± SD	Type of the diabetes (% *n*)	Techniques	Geography
Olaleye et al., 2001 [25]	11.5 ± NR	Type 1 (17.4%)	Yes-no	Canada
Perkins et al., 2001 [26]	12.53 ± 11.47	Type 1 (17.4%)	Yes-no	Canada
		Type 2 (69.7%)		
		NGT (12.9%)		
Lee et al., 2003 [27]	14.8 ± 6.7	Type 2	Yes-no	Korea
Mythili et al., 2010 [28]	6.9 ± NR	Type 2	Yes-no	India
Perkins et al., 2010 [29]	13 ± 9	Type 2 (84%)	Forced choice (0, 0.5, 1)	Canada
Pambianco et al., 2011 [30]	33.6 ± 5.2(*n* = 25)	Type 1	Yes-no	USA
	38.3 ± 7.2(*n* = 170)			
Pourhamidi et al., 2014 [31]	7.2 ± 0.9	NGT (33%)	Unknown	Sweden
		IGT (24%)		
		Type 2 (43%)		
Baraz et al., 2014 [32]	6.1 ± 7.7	Type 2	Yes-no & point the site	Iran
Ruhdorfer et al., 2015 [33]	12.2 ± 10.3	Type 1 (7.3%)	Yes-no	Austria
		Type 2 (92.7%)		

NGT: normal glucose tolerance; IGT: impaired glucose tolerance; NR: not reported in the paper.

**Table 4:** Meta-analysis of diagnostic accuracy under the HSROC model.

	Pooled value	SE	95% CI	
Sensitivity	0.53	0.12	0.32	0.74
Specificity	0.88	0.04	0.78	0.94
DOR	8.62	2.68	4.69	15.84
LR+	4.56	1.03	2.93	7.10
LR−	0.53	0.11	0.35	0.81

SE: standard error; CI: confidence interval.
