# Supplementary material for: Diagnostic Accuracy of Monofilament Tests for Detecting Diabetic Peripheral Neuropathy: A Systematic Review and Meta-Analysis
Source: J Diabetes Res. 2017 Oct 8;2017:8787261. doi: 10.1155/2017/8787261 (PMC5651135; doi:10.1155/2017/8787261)
Supplement: Supplementary file 1 — Appendix 1 Search strategy for EMBASE (OvidSP). Appendix 2 Search strategy for MEDLINE (OvidSP). Appendix 3 Search strategy for Cochrane. Appendix 4 Search strategy for Web of Science. Appendix 5 Assessment of methodological quality table QUADAS-2 tool. [file 8787261.f1.pdf]

Appendix 1 Search strategy for EMBASE (OvidSP)

Database: Embase <1974 to 2016 April 08> Search Strategy:

1 exp Diabetes Mellitus/ (710754)
2 diabet*.mp. (844729)
3 1 or 2 (848720)
4 peripheral nervous.mp. (72715)
5 disorder.mp. (1383912)
6 exp Disease/ (18789225)
7 4 and 5 (4310)
8 4 and 6 (47282)
9 exp Peripheral Nervous System Diseases/ (56273)
10 exp Polyneuropathies/ (31581)
11 Polyneuropath*.mp. (24089)
12 neuropath*.mp. (244404)
13 7 or 8 or 9 or 10 or 11 or 12 (300966)
14 3 and 13 (41329)
15 Diabetic Neuropathies/ (11484)
16 14 or 15 (41329)
17 exp Diagnosis/ (5385929)
18 exp "Sensitivity and Specificity"/ (243304)
19 diagnostic accuracy.mp. (210841)
20 accuracy.mp. (581427)
21 screening test.mp. (67885)
22 sensitivity.mp. (1070529)
23 specificity.mp. (687896)
24 17 or 18 or 19 or 20 or 21 or 22 or 23 (6629021)
25 semmes weinstein.mp. (824)
26 monofilament test.mp. (224)
27 exp Nylons/ (4834)
28 filament.mp. (45283)
29 pressure sensation.mp. (483)
30 pin prick sensation.mp. (61)
31 25 or 26 or 27 or 28 or 29 or 30 (51439)
32 16 and 24 and 31 (173)

Appendix 2 Search strategy for MEDLINE (OvidSP)

Database: Ovid MEDLINE(R) 1946 to Present with Daily Update, Ovid MEDLINE(R) In-Process & Other Non-Indexed Citations <April 08, 2016> Search Strategy:

1 exp Diabetes Mellitus/ (345596)
2 diabet*.mp. (529653)
3 1 or 2 (531161)
4 peripheral nervous.mp. (38606)
5 disorder.mp. (496358)
6 exp Disease/ (174671)
7 4 and 5 (1600)
8 4 and 6 (839)
9 exp Peripheral Nervous System Diseases/ (126905)
10 exp Polyneuropathies/ (23656)
11 Polyneuropath*.mp. (14276)
12 neuropath*.mp. (113117)
13 7 or 8 or 9 or 10 or 11 or 12 (199693)
14 3 and 13 (23012)
15 Diabetic Neuropathies/ (12812)
16 14 or 15 (23012)
17 exp Diagnosis/ (7046725)
18 exp "Sensitivity and Specificity"/ (466573)
19 diagnostic accuracy.mp. (28822)
20 accuracy.mp. (270820)
21 screening test.mp. (16698)
22 sensitivity.mp. (893823)
23 specificity.mp. (887511)
24 17 or 18 or 19 or 20 or 21 or 22 or 23 (7989169)
25 semmes weinstein.mp. (658)
26 monofilament test.mp. (152)
27 exp Nylons/ (4611)
28 filament.mp. (29825)
29 pressure sensation.mp. (161)
30 pin prick sensation.mp. (30)
31 25 or 26 or 27 or 28 or 29 or 30 (35258)
32 16 and 24 and 31 (125)

Appendix 3 Search strategy for Cochrane

Cochrane Library Search Manager:
#1 MeSH descriptor Diabetes mellitus explode all trees
#2 diabet*:ti,ab,kw (Word variations have been searched)
#3 #1 or #2
#4 peripheral nervous:ti,ab,kw (Word variations have been searched)
#5 disorder:ti,ab,kw (Word variations have been searched)
#6 disease:ti,ab,kw (Word variations have been searched)
#7 #4 and #5
#8 #4 and #6
#9 MeSH descriptor: [Peripheral Nervous System Diseases] explode all trees
#10 MeSH descriptor: [Polyneuropathies] explode all trees
#11 Polyneuropath*:ti,ab,kw (Word variations have been searched)
#12 neuropath*:ti,ab,kw (Word variations have been searched)
#13 #7 or #8 or #9 or #10 or #11 or #12
#14 #3 and #13
#15 MeSH descriptor: [Diabetic Neuropathies] explode all trees
#16 #14 or #15
#17 MeSH descriptor: [Diagnosis] explode all trees
#18 MeSH descriptor: [Sensitivity and Specificity] explode all trees
#19 diagnostic accuracy:ti,ab,kw (Word variations have been searched)
#20 accuracy:ti,ab,kw (Word variations have been searched)
#21 screening test:ti,ab,kw (Word variations have been searched)
#22 sensitivity:ti,ab,kw (Word variations have been searched)
#23 specificity:ti,ab,kw (Word variations have been searched)
#24 #17 or #18 or #19 or #20 or #21 or #22 or #23
#25 semmes weinstein:ti,ab,kw (Word variations have been searched)
#26 monofilament test:ti,ab,kw (Word variations have been searched)
#27 nylon:ti,ab,kw (Word variations have been searched)
#28 filament:ti,ab,kw (Word variations have been searched)
#29 pressure sensation:ti,ab,kw (Word variations have been searched)
#30 pin prick sensation:ti,ab,kw (Word variations have been searched)
#31 #25 or #26 or #27 or #28 or #29 or #30
#32 #16 and #24 and #31

Appendix 4 Search strategy for Web of Science

# 8	191	#7 AND #5 AND #4<br>Indexes=SCI-EXPANDED, SSCI, A&HCI, CPCI-S, CPCI-SSH, ESCI, CCR-EXPANDED, IC Timespan=All years
# 7	17,226	#6 AND #3<br>Indexes=SCI-EXPANDED, SSCI, A&HCI, CPCI-S, CPCI-SSH, ESCI, CCR-EXPANDED, IC Timespan=All years
# 6	200,652	#2 AND #1<br>Indexes=SCI-EXPANDED, SSCI, A&HCI, CPCI-S, CPCI-SSH, ESCI, CCR-EXPANDED, IC Timespan=All years
# 5	119,900	TOPIC: (semmes weinstein OR monofilament test OR nylons OR filament OR pressure sensation OR pin prick sensation)<br>Indexes=SCI-EXPANDED, SSCI, A&HCI, CPCI-S, CPCI-SSH, ESCI, CCR-EXPANDED, IC Timespan=All years
# 4	2,974,845	TOPIC: (Diagnosis OR Sensitivity and Specificity OR diagnostic accuracy OR accuracy OR screening test OR sensitivity OR specificity)<br>Indexes=SCI-EXPANDED, SSCI, A&HCI, CPCI-S, CPCI-SSH, ESCI, CCR-EXPANDED, IC Timespan=All years
# 3	17,226	TOPIC: (Diabetic Neuropathies)<br>Indexes=SCI-EXPANDED, SSCI, A&HCI, CPCI-S, CPCI-SSH, ESCI, CCR-EXPANDED, IC Timespan=All years
# 2	3,255,257	TOPIC: (Peripheral Nervous System Diseases OR Polyneuropathies OR Polyneuropath* OR neuropath*) OR TOPIC: (peripheral nervous OR disease) OR TOPIC: (peripheral nervous AND disorder)<br>Indexes=SCI-EXPANDED, SSCI, A&HCI, CPCI-S, CPCI-SSH, ESCI, CCR-EXPANDED, IC Timespan=All years
# 1	549,625	TOPIC: (Diabetes Mellitus OR diabet*)<br>Indexes=SCI-EXPANDED, SSCI, A&HCI, CPCI-S, CPCI-SSH, ESCI, CCR-EXPANDED, IC Timespan=All years

Appendix 5 Assessment of methodological quality table QUADAS-2 tool

DOMAIN	PATIENT SELECTION	INDEX TEST	REFERENCE STANDARD	FLOW AND TIMING
Description	Describe methods of patient selection: Describe included patients (prior testing, presentation, intended use of index test and setting):	Describe the index test and how it was conducted and interpreted	Describe the reference standard and how it was conducted and interpreted	Describe any patients who did not receive the index test(s) and/or reference standard or who were excluded from the 2x2 table (refer to flow diagram): Describe the time interval and any interventions between index test(s) and reference standard
Signaling questions<br><br>(yes/no/unclear)	Was a consecutive or random sample of patients enrolled?	Were the index test results interpreted without knowledge of the results of the reference standard?	Is the reference standard likely to correctly classify the target condition?	Was there an appropriate interval between index test(s) and reference standard?
	Was a case-control design avoided?	If a threshold was used, was it pre-specified?	Were the reference standard results interpreted without knowledge of the results of the index test?	Did all patients receive a reference standard?
	Did the study avoid inappropriate exclusions?			Did all patients receive the same reference standard?
				Were all patients included in the analysis?
Risk of bias: High/low/ unclear	Could the selection of patients have introduced bias?	Could the conduct or interpretation of the index test have introduced bias?	Could the reference standard, its conduct, or its interpretation have introduced bias?	Could the patient flow have introduced bias?
Concerns regarding applicability: High/low/ unclear	Are there concerns that the included patients do not match the review question?	Are there concerns that the index test, its conduct, or interpretation differ from the review question?	Are there concerns that the target condition as defined by the reference standard does not match the review question?	
